# Case report: A choroidal fissure pial arteriovenous malformation inducing venous congestive edema of the medulla oblongata and cervicothoracic spinal cord presented with proximal arm predominant weakness

**DOI:** 10.3389/fneur.2023.1128366

**Published:** 2023-05-16

**Authors:** Yun Jiang, Ying Zhou, Ximeng Yang, Aizhen Sheng, Jun Lu

**Affiliations:** ^1^Department of Neurology, Beijing Hospital, National Center of Gerontology, Institute of Geriatric Medicine, Chinese Academy of Medical Sciences, Beijing, China; ^2^Department of Geriatrics, The First People's Hospital of Yunnan Province, Kunming, China; ^3^Department of Neurosurgery, Beijing Hospital, National Center of Gerontology, Institute of Geriatric Medicine, Chinese Academy of Medical Sciences, Beijing, China

**Keywords:** choroidal fissure, intracranial arteriovenous malformation, myelopathy, interventional treatment, intracranial dural arteriovenous fistula

## Abstract

Intracranial dural arteriovenous fistula (DAVF) can induce remote myelopathy *via* spinal perimedullary venous drainage. In the present study, we report a rare case of intracranial pial arteriovenous malformation (AVM)-related myelopathy. A 52-year-old man presented with progressive, predominantly proximal weakness and muscle atrophy in bilateral upper limbs, urinary retention, and hyperreflexia in bilateral upper and lower limbs. Brain and cervicothoracic MRI showed longitudinal myelopathy extending from the medulla oblongata to the T6 level, with perimedullary enlarged veins from the C1 to T12 level, and remarkable enhancement in bilateral anterior horns from the C2 to C7 level. Cerebral angiography revealed a choroidal fissure AVM, which was supplied by the left anterior choroidal artery and drained exclusively by an inferior ventricular vein descending toward the spinal perimedullary veins. After endovascular embolization of the feeding pedicle, nidus, and proximal segment of the draining vein, the patient's neurological deficits rapidly improved, and a significant recovery was achieved 3 months after the procedure. This rare case indicates that intracranial pial AVM can also cause extensive congestive myelopathy with similar mechanisms underlying intracranial and craniocervical DAVF cases, and gray matter in the spinal cord might be more susceptible to ischemia induced by intraspinal venous hypertension.

## Highlights

- Intracranial pial AVM with spinal venous drainage can cause extensive congestive myelopathy.
- Contrasted MRI images suggest that the gray matter of the spinal cord might be more susceptible to ischemia caused by intraspinal venous hypertension.

## Introduction

A series of case studies have demonstrated that intracranial dural arteriovenous fistula (DAVF) with spinal venous drainage can cause extensive myelopathy (1–4). Most of them are infratentorial, and few are supratentorial (3, 5). The supratentorial DAVF was drained downward into the perimesencephalic veins, the superior petrosal veins, and finally the anterior and posterior spinal veins (3, 6). Here, we present a rare case of a choroidal fissure pial arteriovenous malformation (AVM) with exclusive spinal venous drainage that induced venous congestive edema of the medulla oblongata and the cervicothoracic spinal cord. To the best of our knowledge, only one case of infratentorial trigeminal nerve root pial AVM-associated myelopathy has been reported, whereas no cases of supratentorial pial AVM-causing myelopathy have been described (7). In contrast to the intracranial DAVF, in which patients mainly present with ascending myelopathy (1–5), our patient presented with pronounced arm weakness with minimal leg involvement 2 months after the onset of symptoms. The likely underlying mechanisms of this unusual manifestation are discussed further in the article.

## Case presentation

A previously healthy 52-year-old man initially presented with fluctuating neck tightness and predominantly proximal weakness in bilateral arms, which worsened at night and almost disappeared in the morning. Within 1 month, his symptoms became persistent and caused a deterioration in his activities. He also developed urinary retention. He was admitted to our hospital approximately 2 months after the onset of symptoms when he could not dress himself. Neurological examination revealed muscular atrophy of bilateral proximal upper limbs. Based on the Medical Research Council (MRC) scale, muscle strength was graded 2/5 on shoulders, 3/5 in proximal and 4/5 in distal upper limbs, respectively, and 5/5 in the left and normal in the right lower limb. There was hyperreflexia in four extremities, with clonus present in both ankles. There were no signs of pathological reflexes nor sensory deficits, ataxia, dysarthria, or dysphagia.

**Figure 1:**
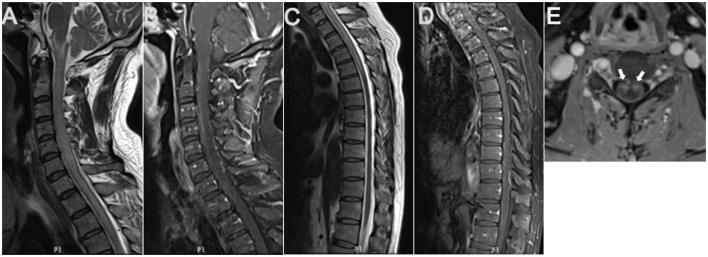
Cervicothoracic MRI. Sagittal cervicothoracic MRI T2-weighted images revealed longitudinal swelling of the medulla oblongata and cervicothoracic spinal cord extending to T6 level **(A, C)**, with apparent abnormal flow void signals in the anterior surface of the cervical spinal cord **(A)**. Contrasted MRI showed anterior and posterior perimedullary dilated tortuous veins from the cervical to T12 level **(B, D)**, apparent enhancement in cervical spinal anterior horns on both sides (arrows) **(E)**, and patchy enhancement in the whole cervicothoracic spinal cord **(B, D)**.

**Figure 2:**
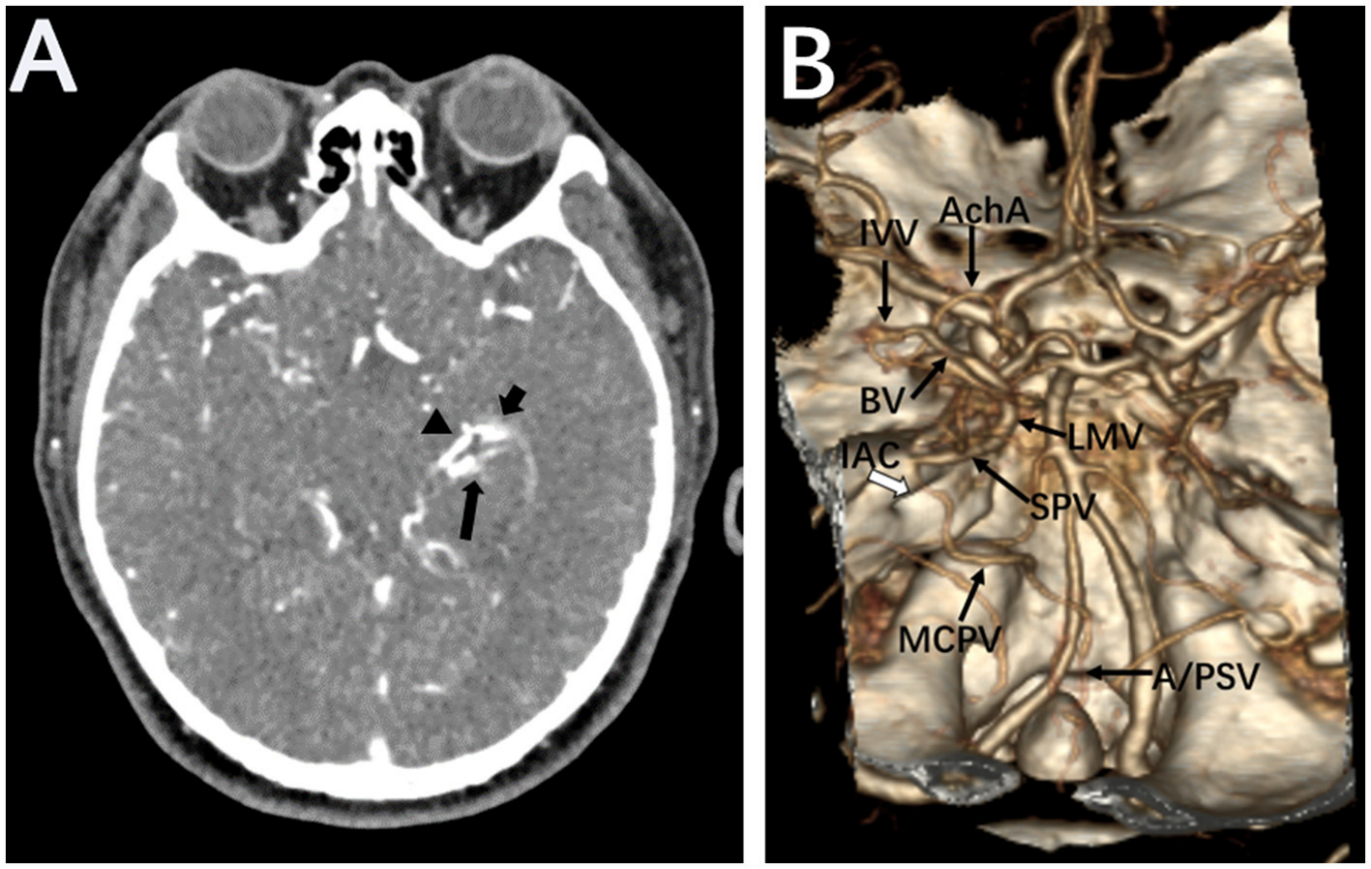
Brain CTA. Source images of brain CTA showed a large abnormal vessel (arrowhead) connecting to a vascular cluster close to the inferior horn of the left lateral ventricle (short arrow). The left posterior cerebral artery was also disclosed (long arrow) **(A)**. Volume reconstruction of CTA revealed the supplied artery and drainage veins of the micro-AVM on the choroidal fissure **(B)**. anterior choroidal artery, AchA; inferior ventricular vein, IVV; basal vein of Rosenthal, BA; lateral mesencephalic vein, LMV; superior petrosal vein, SPV; middle cerebellar peduncle vein, MCPV; anterior and posterior spinal veins, A/PSV; internal auditory canal, IAC.

**Figure 3:**
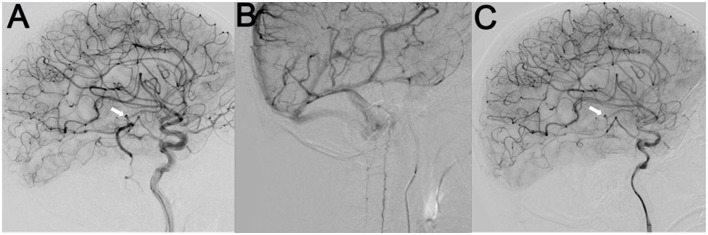
Cerebral angiography indicated a micro-AVM (arrow) supplied by the left anterior choroidal artery branch, which drained downward through a sole large vein into the anterior and posterior spinal veins **(A, B)**. After the endovascular embolization of the AVM, the draining veins completely disappeared **(C)**.

Blood and cerebral spinal fluid (CSF) routine and immunological tests were unremarkable, except for a slightly increased CSF protein level of 627.5 mg/L (normal range: 150–450 mg/L). Magnetic resonance imaging (MRI) revealed extensive longitudinal swelling of the medulla oblongata and cervicothoracic spinal cord with enlarged abnormal perimedullary tortuous vessels and notable enhancement in bilateral cervical anterior gray matter (Figures 1A–E). Source images of the brain CT angiogram showed a large abnormal vessel connected to a vascular cluster adjacent to the inferior horn of the left lateral ventricle (Figure 2A). 3D volume reconstructing images of brain CT angiogram (Figure 2B) and cerebrospinal angiography (Figures 3A, B) showed a micro-AVM on the choroidal fissure close to the left cerebral ventricular wall, which was supplied by the left anterior choroidal artery and drained exclusively by an inferior ventricular vein, connecting to the basal vein of Rosenthal, the lateral mesencephalic vein, the superior petrosal vein, then the middle cerebellar peduncle vein, and finally the anterior and posterior spinal veins, descending toward lower thoracic spinal cord (Supplementary Videos 1, 2). The left superior petrous sinus was considered to be occluded since it was not opacified in carotid and vertebral angiographies.

The AVM was treated by endovascular embolization using 18% diluted N-butyl-2-cyanoacrylate (NBCA). The feeding pedicle, nidus, and proximal segment of the draining vein were completely obliterated (Figure 3C).

After the procedure, his neck tightness significantly improved, and he was able to lift his arms and dress himself within 1 week. After 2 months, his upper limb weakness had almost completely resolved, with hyperreflexia still present in the upper and lower limbs. MRI follow-up showed a marked decrease in T2 hyperintensity in the spinal cord as well as the resolution of the abnormal perimedullary flow voids (Supplementary Figures 1A, B). His urinary retention was completely resolved 3 months after the procedure. An angiographic follow-up showed complete obliteration of the AVM (Supplementary Videos 3, 4). An MRI follow-up 9 months after the procedure showed a complete disappearance of T2 hyperintensity in the cervical spinal cord (Supplementary Figure 1C).

## Discussion

Similar to those shown in intracranial DAVF cases with spinal venous drainage (1–6), cervicothoracic MRI in the current case showed venous congestive edema of the medulla oblongata and spinal cord, as well as perimedullary dilated tortuous vessels. Cerebrospinal angiography revealed a choroidal fissure pial micro-AVM with spinal venous drainage, which was considered to be the cause of extensive myelopathy. To the best of our knowledge, this is the first case of supratentorial pial AVM which leads to remote myelopathy.

Shimizu et al. (8) reported one case of anterior cranial fossa DAVF causing venous congestion of the brain stem and spinal cord. The shunt drained into the olfactory vein, basal vein, lateral mesencephalic vein, superior petrous vein, and spinal veins. The superior petrous sinus was occluded. Our case revealed a similar venous drainage route, except that the pial arteriovenous shunt initially drained to an inferior ventricular vein and then to the basal vein of Rosenthal. The superior petrous sinus was also occluded in our case. Surgical anatomic studies have demonstrated that lateral mesencephalic veins connect the basal vein to the superior petrosal vein/sinus, which may permit venous drainage from supratentorial structures directly into the superior petrosal sinus (9). We postulate that the anastomosis role of the lateral mesencephalic vein and occlusion of the superior petrosal sinus are the key factors for the exclusive spinal drainage of this supratentorial AVM case. This rare case has helped us gain insight into a unique mechanism associated with intracranial pial arteriovenous shunt-related congestive myelopathy.

Intracranial and spinal arteriovenous shunts with spinal venous drainage can lead to hypertension in the spinal perimedullary venous plexus and then in the spinal intramedullary veins (2, 3, 6). Consequently, the intraspinal arteriovenous pressure gradient is reduced, resulting in decreased tissue perfusion. Edema, ischemia, degeneration, and necrosis of the spinal cord, and rarely subarachnoid hemorrhage, may develop (10). In several autopsies, severe dilation of perimedullary and intraspinal veins and arteries as well as gray matter-predominant necrosis was observed. In line with the abovementioned reports, the contrasted cervical MRI of our case also revealed a significant enhancement of bilateral gray matter. These findings indicate that spinal vein hypertension plays a key role in this process and that gray matter is more susceptible to ischemia when compared to white matter (11).

In our case, the pial AVM-induced longitudinal myelopathy mainly caused symptoms in the upper limbs. The spectrum of the clinical manifestation, in this case, is different from that in most cranial or spinal DAVF-related myelopathy cases, which tend to cause more pronounced weakness in the lower limbs than the upper ones (1, 3, 5, 6). In addition to the diffuse, patchy enhancement of the spinal cord, contrasted MRI showed the focal enhancement of bilateral cervical anterior gray matter, which may explain his symptoms of arm predominant weakness. The potential mechanism needs further investigation.

## Conclusion

Pial AVM can be a rare cause of extensive congestive myelopathy, with similar mechanisms underlying those reported cases of cranial DAVF-associated myelopathy. Gray matter in the spinal cord may be susceptible to ischemia induced by intraspinal venous hypertension. The complete obliteration of the arteriovenous shunt before irreversible damage occurs is crucial for a favorable prognosis.

## Data availability statement

The original contributions presented in the study are included in the article/Supplementary material, further inquiries can be directed to the corresponding author.

## Ethics statement

The studies involving human participants were reviewed and approved by the Ethics Committee of the Beijing Hospital. The patients/participants provided their written informed consent to participate in this study. Written informed consent was obtained from the patient for the publication of any potentially identifiable images or data included in this article.

## Author contributions

YJ and JL instructed the patient's diagnosis and treatments and followed the patient. YJ drafted the manuscript. YZ, XY, and AS assisted in the patient's diagnosis and treatment. All authors contributed to the article and approved the submitted version.
