# Supplementary figures and images for: Case report: A choroidal fissure pial arteriovenous malformation inducing venous congestive edema of the medulla oblongata and cervicothoracic spinal cord presented with proximal arm predominant weakness
Source: Front Neurol. 2023 May 16;14:1128366. doi: 10.3389/fneur.2023.1128366 (PMC10227433; doi:10.3389/fneur.2023.1128366)

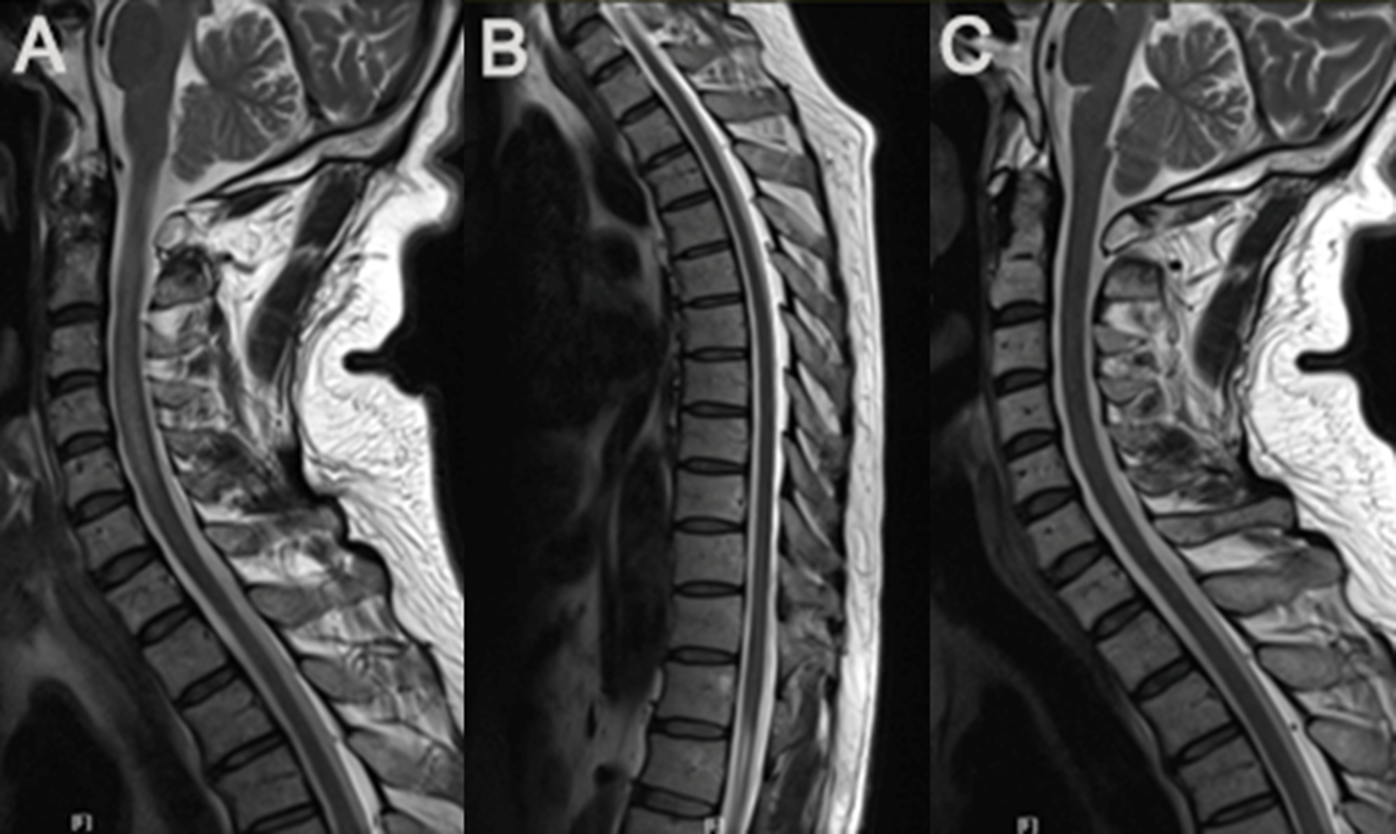

Supplement: Supplementary file 5 [file Image_1.TIF]
